# Efficacy and safety of platelet-rich plasma in the treatment of carpal tunnel syndrome: A network meta-analysis of different injection treatments

**DOI:** 10.3389/fphar.2022.906075

**Published:** 2022-11-10

**Authors:** Pan Hong, Yu Zheng, Saroj Rai, Yuhong Ding, Yeming Zhou, Ruikang Liu, Jin Li

**Affiliations:** ^1^ Department of Orthopaedic Surgery, Union Hospital, Tongji Medical College, Huazhong University of Science and Technology, Wuhan, China; ^2^ Basic Medical School, Tongji Medical College, Huazhong University of Science and Technology, Wuhan, China; ^3^ Department of Orthopaedics and Trauma Surgery, Karama Medical Center, Dubai, United Arab Emirates; ^4^ Second Clinical School, Tongji Medical College, Huazhong University of Science and Technology, Wuhan, China; ^5^ Department of Endocrinology, Union Hospital, Tongji Medical College, Huazhong University of Science and Technology, Wuhan, China

**Keywords:** carpal tunnel syndrome, platelet-rich plasma, corticosteroid, network meta-analysis, estrogen

## Abstract

**Purpose:** Carpal tunnel syndrome (CTS) is a common form of median nerve compression in the wrist caused by focal peripheral neuropathy. Platelet-rich plasma (PRP) therapy could improve the healing ability by exposing the injured tissues to high concentrations of autologous growth factors. Our study aims to compare all injective treatments for CTS and assess the efficacy and priority of PRP therapy.

**Methods:** We searched Medline, Embase, Web of Science, Cochrane databases, and Clinicaltrial.gov until 17 October 2022. We only included data from randomized controlled trials (RCTs) that evaluated PRP injection therapy or drug injection therapy. The included RCTs measured at least one of the following three outcomes with validated instruments: in the visual analog scale (VAS), symptom severity scale (SSS), and functional status scale (FSS).

**Results:** Overall, 19 studies with 1,066 patients were included in this study. We used the SUCRA rankings to determine the merits of various therapies. In all, 5% dextrose injections were the best treatment strategy for the VAS (MD −1.22, 95% CI −2.66 to 0.23; SUCRA = 79.2%), followed by triamcinolone (high-dose) injections (MD −0.69, 95% CI −2.11 to 0.73; SUCRA = 62.7%) and PRP injections (MD −0.39, 95% CI −1.67 to 0.89; SUCRA = 60.0%). In the SSS, the most effective intervention was hydroxyprogesterone injections (MD −0.62, 95% CI −1.09 to −0.16; SUCRA = 91.0%). The SUCRA ranking of PRP was second only to steroids and estrogen (MD −0.39, 95% CI −0.60 to −0.18; SUCRA = 60.8%). In the FSS, the best regimen strategy was hydroxyprogesterone injections (MD 0.12, 95% CI −0.30 to 0.54; SUCRA = 99.5%), followed by triamcinolone (low-dose) injections (MD −0.02, 95% CI −0.54 to 0.49; SUCRA = 87.4%) and PRP injections (MD −0.26, 95% CI −0.43 to −0.09; SUCRA = 77.1%).

**Conclusion:** PRP is an alternative choice for CTS treatment. PRP injection is second only to steroids and estrogen in the treatment efficacy of CTS, with a wide indication and safe outcome.

## Introduction

Carpal tunnel syndrome (CTS) is a common form of median nerve compression in the wrist caused by focal peripheral neuropathy. CTS causes impaired nerve conduction, resulting in symptoms such as pain, burning, tingling, or abnormal pain and sensation in the wrist (Sternbach, 1999; Katz and Simmons, 2002; Aroori and Spence, 2008). Common causes of CTS include trauma, inflammation, obesity, occupational exposure, older age, and pregnancy (Zamborsky et al., 2017). The prevalence of CTS is strongly associated with age and sex, and women over 50 years old were reported as the highest risk group (Tanaka et al., 1994; Bland and Rudolfer, 2003). The total lifetime prevalence was estimated up to 6.7% in the US worker population (Atroshi et al., 1999).

Nowadays, the mainstream pharmacological therapy for CTS is the injection of corticosteroids into the carpal tunnel, to reduce edema and improve the spatial relationship between the median nerve and its surrounding tissue (Karjalanen et al., 2022). However, its pros and cons remain controversial. Atroshi et al. reported in 2013 that three-quarters of patients receiving corticosteroid injections had re-operation within 1 year. Kamanli et al. (2011) also demonstrated that the pain after corticosteroid treatment only reduced in the patient’s subjective (the visual analog scale measure of pain) rather than in objective measures (such as nerve conduction fixation) (Padua et al., 2016). Other injection treatments for CTS include progesterone (Raeissadat et al., 2017a), dextrose solution (Lin et al., 2020), and non-steroidal anti-inflammatory drugs (NSAIDs) (Boonhong and Thienkul, 2020a). However, the efficacy comparison of these treatments had not reached a consensus.

The principle of platelet-rich plasma (PRP) therapy is to improve the healing ability by exposing the injured tissues to high concentrations of autologous growth factors (Dhillon et al., 2012). A series of clinical trials in recent years had shown that PRP infusion could achieve significant benefits and safe results in patients with CTS (Wu et al., 2017a; Malahias et al., 2019). Our network meta-analysis aims to compare all the injective treatments for CTS and assess the efficacy and priority of PRP therapy.

## Methods

### Search strategy

Our review followed the guidelines of Preferred Reporting Items for Systematic Reviews and Meta-Analyses (PRISMA), and the protocol was registered in PROSPERO (registration number CRD42022307089) before the literature search (see Supplementary Table S1). Two independent reviewers (YMZ and RL) searched Medline, Embase, Web of Science, and Cochrane databases updated to 1 February 2022 for randomized controlled trials (RCTs) (we processed another search at the end of the study on 17 October 2022). The search strategy used for the Medline database is available as supplementary material (see Supplementary Table S2). To expand the search range, the keywords were “Carpal tunnel syndrome,” “Platelet-rich plasma,” or “Corticosteroid.” Clinicaltrials.gov was searched for completed but unpublished RCTs. Two researchers (YD and SR) independently screened the titles and abstracts, and articles meeting the inclusion criteria were accessed for full-text review. They independently reviewed full-text articles for eligibility afterward, without language restriction. Reference lists of eligible reviews and trials were searched for additional citations.

### Selection criteria

RCTs with patients diagnosed with CTS by the electrodiagnostic test (including nerve conduction studies), electromyography, imaging, or any other clinical criteria stated by the authors were eligible for inclusion. Considering that treatments with different medication modalities could bring higher heterogeneity, we only included RCTs with injection treatment. RCTs were excluded for patients who had undergone surgery or who also had conditions other than CTS, such as wrist fractures and infections. There were no restrictions on age, sex, nationality, and race. Only RCTs published in English were included. The eligible RCTs included at least one of the following outcomes:

1) Changes in CTS-related pain, measured by the visual analog scale (VAS);
2) Changes in CTS symptom severity, measured by the Boston Carpal Tunnel Syndrome Symptom Severity Scale (SSS);
3) Changes in the CTS functional status, measured by the Boston Carpal Tunnel Syndrome Function Severity Scale (FSS).

Outcome 1) is an important patient-oriented care outcome, while outcomes 2) and 3) were recommended by the American Academy of Orthopaedic Surgeons as instruments for the assessment of CTS treatment (Keith et al., 2009).

### Data extraction

Two researchers (YD and YMZ) independently extracted data from eligible articles. The extracted data included characteristics of the study, characteristics of the patient, and baseline and outcome data. In the case of disagreements and failed consensus, decisions were made by consulting a third reviewer, SR. When data were incomplete, the corresponding author would be contacted by email and invited to send additional information. Outcomes included the changes in the VAS, SSS, and FSS. The first-line therapeutic agents included triamcinolone, methylprednisolone, hydroxyprogesterone, dextrose solutions, ozone, and piroxicam. Triamcinolone and methylprednisolone injection therapies were further divided into different treatments, according to their doses since there was a significant difference in efficacy between the high and low doses. All the assessment tools of the included RCTs are shown in Appendix.

### Quality assessment

The Cochrane risk of bias assessment tool (CROBAT) was used by two researchers (RL and YZ) to independently assess the quality of the included studies. CROBAT included “Random sequence generation,” “Allocation concealment,” “Blinding of participants and personnel,” “Blinding of outcome assessment,” “Incomplete outcome data,” “Selective reporting,” and “Other bias” (Supplementary Table S4). Each question had three answers: “Low-risk,” “Moderate,” and “High-risk.” According to the published information, researchers would assess the risk level of RCTs. The decision was reached by consulting a third reviewer, SR, in the case of disagreements and failed consensus.

### Statistical analysis

We used a network meta-analysis to perform indirect comparisons of the effectiveness of different treatments among the included RCTs for CTS. Review Manager 5.3 and STATA version 13.0 (STATA Corp., College Station, TX, United States) were used in our study. The network meta-analysis was conducted to explore the probability that PRP would be more effective than the other drug treatments evaluated in the included RCTs. Indirect comparisons of continuous outcomes among different treatments were implemented with the *mvmeta* command. The effectiveness ranking of the treatments was computed from the mean difference (continuous outcome) of each possible pair of comparisons. To reflect the rank and uncertainty, we used the surface under the cumulative ranking area (SUCRA) described by Salanti et al. (2011). This measure demonstrated the relative probability of whether the intervention was one of the best choices. We also constructed network diagrams based on the treatment strategies of the included studies.

All statistical tests were two-tailed, and *p* ≤ 0.05 was regarded as a statistically significant difference. There are two types of statistical methods for continuous variables: standard mean difference (SMD) and mean difference (MD). For continuous-type variables of the same range which do not require standardization, the MD is chosen to bring statistical differences with clearer quantitative results directly (results with the unit), whereas SMD is generally used for data on different magnitudes that require standardization (results without the unit), such as comparing the operative time (in hours) and hospitalization time (in days). Therefore, the MD was the applicable method in our meta-analysis. Since all three variables we analyzed were continuous variables, MD with 95 % CI was used for data analyses. Heterogeneity in the result of the meta-analysis was assessed using Cochrane Q and I^2^ statistics with appropriate analysis models. Clinical and methodological heterogeneity were assessed by carefully examining the characteristics and design of the included studies. The major sources of clinical heterogeneity included age and sex. Additionally, we would assess the reporting bias by examining the asymmetry of a funnel plot (Begg, 2002), which is the most common tool used in meta-analyses to assess the presence of small study effects (Chaimani et al., 2013).

Subgroup analysis would be carried out when detailed data were available. We classified the different injection methods by applying the categories described in “Types of Interventions”. For studies with different drug doses, we selected the data set with the most significant effect. Since the effects of methylprednisolone and triamcinolone vary greatly by dose, we divided them into two doses of therapy, large and small, with 40 mg and 20 mg as the dividing line, respectively. Moreover, we did not perform subgroup analyses for different injection techniques and batches of drugs.

## Results

### Study selection

**FIGURE 1:**
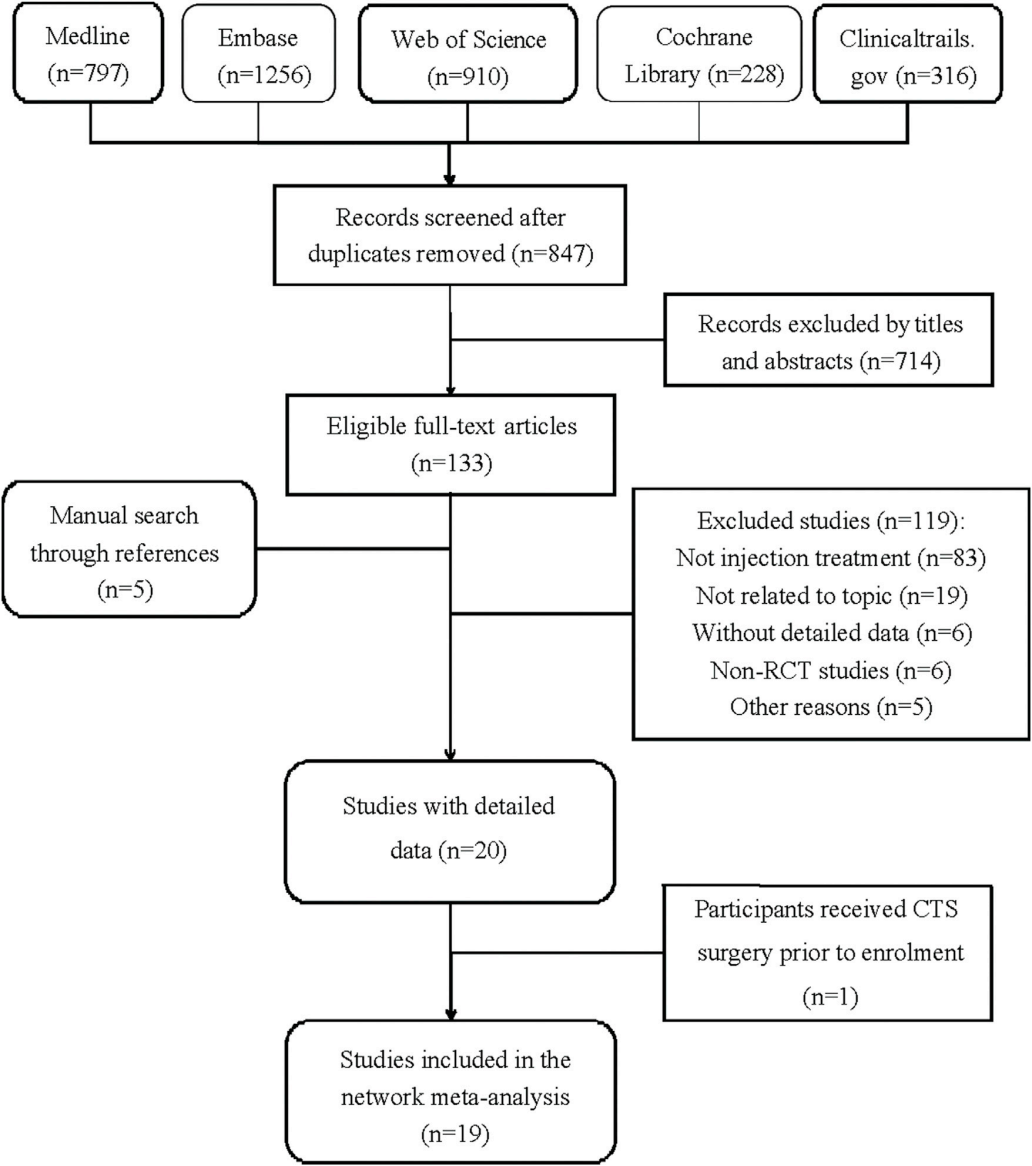
Flowchart of selection of the included studies.

Figure 1 demonstrates the detailed steps of the literature search. Table 1 summarizes the details of the study selection process. After retrieving 847 studies, 714 studies were screened out by browsing the titles and abstracts, and the remaining 133 studies were for full-text reviewing afterward. Subsequently, 121 studies were excluded, and 5 studies were added through searching for references. In all, 18 RCTs met the inclusion criteria (Peters-Veluthamaningal et al., 2010; Ginanneschi et al., 2012; Karadaş et al., 2012; Atroshi et al., 2013; Bahrami et al., 2015; Wu et al., 2017c; Dernek et al., 2017; Uzun et al., 2017; Raeissadat et al., 2018; Wu et al., 2018; Güven et al., 2019; Senna et al., 2019; Shen et al., 2019; Boonhong and Thienkul, 2020b; Hsu et al., 2020; Chen et al., 2021; Forogh et al., 2021). We extracted the outcomes (changes in the visual analog scale, symptom severity scale, and functional status scale) for further data analysis in a short-term (before or near 12 weeks) follow-up period.

**TABLE 1:** Characteristics of the included studies.

Author/Study ID	No.	Treatment (injection)	US.	Injection site	Anesthetic	Age (SD)	Sex (M/F)	Follow-up (weeks)	Funding
Chen 2021/NCT03184688	24	G1: PRP^a^	Yes	Inlet of the carpal tunnel (scaphoid-pisiform level)	No	53.0 (2.0)	3/21	12	None
	24	G2: Placebo				53.0 (2.0)	3/21	12	
Forogh 2021/IRCT20151017024572N5	20	G1: Triamcinolone 40 mg	Yes	N/A	1 ml lidocaine	53.7 (9.3)	N/A	12	Iran University of Medical Sciences
	20	G2: Single ozone (O2-O3)				54.7 (6.6)	N/A	12	
Hsu 2020/NCT03072290	28	G1: Triamcinolone 40 mg	Yes	Inlet of the carpal tunnel (scaphoid-pisiform level)	1 ml lidocaine	57.1 (1.9)	7/21	12	Taipei Veterans General Hospital
	28	G2: Triamcinolone 20 mg				54.5 (1.4)	6/22	12	
Boonhong 2019/RA 57/114	17	G1: Piroxicam 20 mg	Yes	Skin at the carpal tunnel area on the palm side of the hand	No	52.1 (9.8)	1/16	4	King Chulalongkorn Memorial Hospital
	17	G2: Dexamethasone sodium phosphate 60 mg				51.4 (10.6)	0/17	4	
	16	G3: Placebo				51.1 (11.6)	1/15	4	
Güven 2019/E‐14‐267	20	G1: PRP	Yes	Inlet of the carpal tunnel	No	47.5 (15.5)	1/17	4	None
	20	G2: Placebo				50.0 (6.0)	1/11	4	
Senna 2019/NCT03863873	43	G1: PRP	Yes	Inlet of the carpal tunnel	No	38.3 (6.4)	8/35	12	None
	42	G2: Methylprednisolone 40 mg				40.7 (9.4)	6/36	12	
Shen 2019/NCT02696161	26	G1: PRP (3 cc)	Yes	Inlet of the proximal carpal tunnel (scaphoid-pisiform level)	No	56.8 (1.7)	1/25	12	None
	26	G2: 5% dextrose				58.5 (2.1)	4/22	12	
Raeissadat 2018/IRCT2017041513442N13	21	G1: PRP (1 cc)	No	Distal carpal skin crease ulnar side to the palmaris longus tendon	0.5 ml lidocaine	51.2 (9.8)	0/21	10	Clinical development research center of Shahid Modarres hospital
	20	G2: Placebo				47.2 (7.2)	0/20	10	
Wu 2018/NCT02990962	27	G1: 5% dextrose	Yes	Inlet of the carpal tunnel (scaphoid-pisiform level)	No	58.6 (2.2)	5/22	12	Tri-Service General Hospital
	27	G2: Triamcinolone 30 mg				54.3 (2.0)	6/21	12	
Raeissadat 2017/N/A	39	G1: Triamcinolone 20 mg	No	1 cm proximal to wrist crease between the tendons of palmaris longus and flexor carpi radialis	0.5 ml lidocaine	51.0 (8.9)	0/39	10	None
	39	G2: Hydroxy progesterone				47.0 (7.8)	0/39	10	
Uzun 2017/N/A	20	G1: PRP (1 cc)	No	1 cm proximal to the distal wrist crease, ulnar side of the palmaris longus tendon	No	N/A	N/A	12	None
	20	G2: Triamcinolone 40 mg				N/A	N/A	12	
	30	G2: Placebo				58.1 (1.9)	6/24	12	
Wu 2017/NCT02539186	30	G1: PRP	Yes	Inlet of the proximal carpal tunnel (pisiform level)	No	57.9 (1.5)	3/27	12	None
	30	G2: Placebo				54.3 (1.3)	5/25	12	
Wu 2017/NCT02809261	30	G1: 5% dextrose	Yes	Inlet of the carpal tunnel (scaphoid-pisiform level)	No	58.5 (2.3)	4/26	12	None
	30	G2: Placebo				58.1 (1.9)	6/24	12	
Dernek 2016/N/A	38	G1: Betamethasone	No	Ulnar side of the palmaris longus tendon	0.5 cc lidocaine	48.9 (12.4)	2/36	4	None
	29	G2: Placebo				50.5 (12.7)	1/28	4	
Bahrami 2015/IRCT2013101313442N4	30	G1: Triamcinolone 20 mg	Yes	N/A	0.5 ml lidocaine	51.7 (9.7)	0/30	10	None
	30	G2: Hydroxy progesterone				48.2 (9.8)	0/30	10	
Atroshi 2013/NCT00806871	37	G1: Methylprednisolone 80 mg	No	1 cm proximal to the wrist crease, ulnar to midline	1 ml lidocaine	47.0 (12.0)	11/26	10	Region of Scania Research and Development Foundation
	37	G2: Methylprednisolone 40 mg				44.0 (11.0)	10/27	10	
	37	G3: Placebo				49.0 (11.0)	9/28	10	
Karadaş 2012/N/A	20	G1: Triamcinolone 40 mg	No	1 cm proximal to the distal wrist crease, between the palmaris longus and radial flexor tendons	4 ml procaine HCl	46.4 (11.6)	3/17	8	None
	19	G2: Placebo				48.4 (12.1)	2/17	8	
Ginanneschi 2012/N/A	8	G1: Triamcinolone 20 mg	Yes	N/A	No	47 (5.2)	0/8	6	None
	8	G2: Hydroxy progesterone				47 (5.2)	0/8	6	
Peters–Veluthamaningal 2010/N/A	36	G1: Triamcinolone 10 mg	No	Ulnar side of the palmaris longus tendon near the wrist crease	No	56.5 (15.1)	9/27	12	University Medical Center Groningen
	33	G2: Placebo				57.6 (40.3)	7/26	12	

N/A, not applicable; G1, group 1; G2, group 2.

No., number of treated hands.

US., ultrasound.

^a^ PRP, platelet-rich plasma.

### Study description

Table 1 summarizes the detailed characteristics of the included studies. We summarized 19 RCTs with 1,066 patients. Among these RCTs, nine involved triamcinolone (Thd or Tld), six involved platelet-rich plasma (Prp), three involved dextrose (Dex), and others involved methylprednisolone (Mhd or Mld), hydroxyprogesterone (Hyd), betamethasone (Bet), dexamethasone sodium phosphate (Dsp), piroxicam (Pir), and single ozone (Soz). The included RCTs were published between 2010 and 2021. The change in the symptom severity scale was divided by 11 items in the symptom severity scale of the Boston Carpal Tunnel Questionnaire, and the change in the functional status scale was divided by 8 items in the functional status scale of the Boston Carpal Tunnel Questionnaire. In addition, for trials with several different follow-up periods, we only chose to evaluate outcomes that were less than 12 weeks.

**FIGURE 2:**
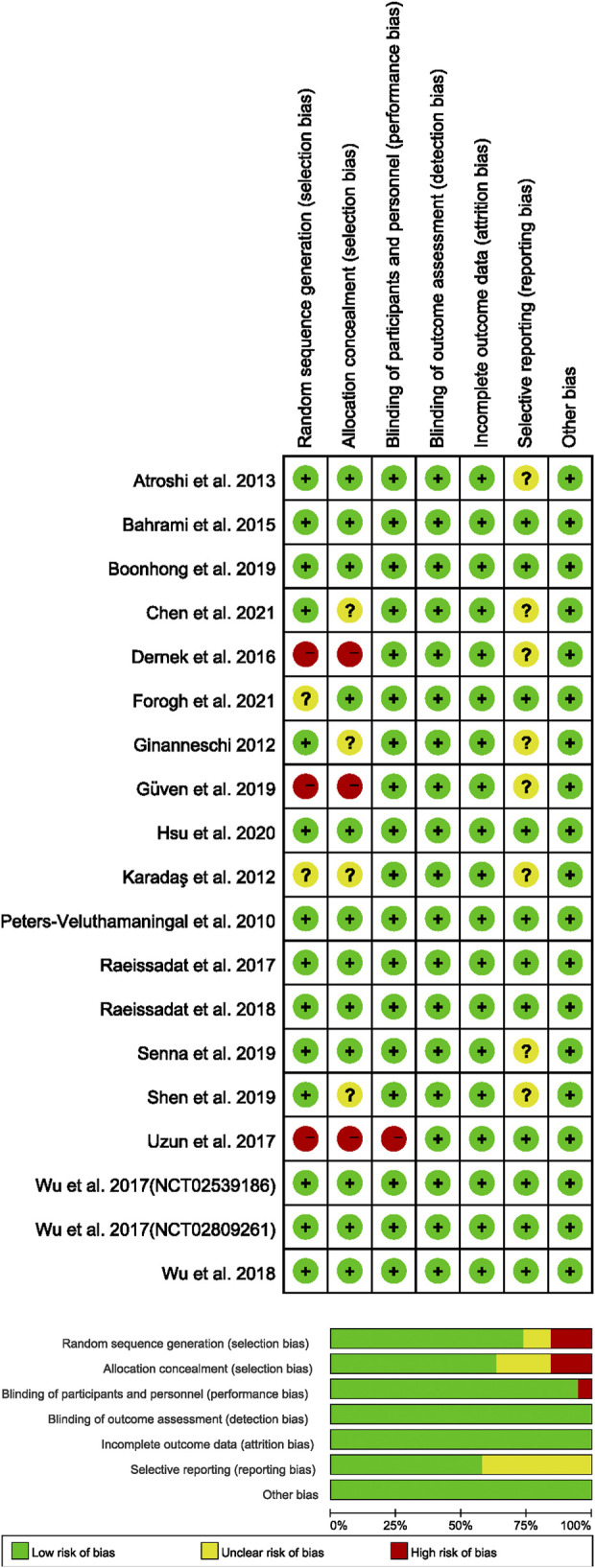
Risk of bias summary. Risk of bias graph.

Supplementary Tables S1, S2 show the low heterogeneity of our data in terms of both age and gender. Figure 2 displays the quality of the included studies. Most of the included RCTs had been described as randomized and concealed allocation.

### Network meta-analysis

**FIGURE 3:**
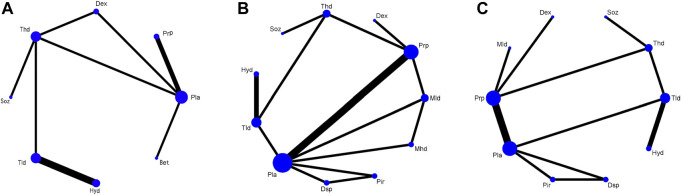
Network plots of the VAS **(A)**, SSS **(B),** and FSS **(C)**.

Figure 3 shows the network plots of the treatments for the changes in the VAS, SSS, and FSS at the 12-week follow-up period. Any two nodes connected by the line represented direct comparisons in the trials. The thickness of the line was proportional to the number of comparisons included in the network, and the width of the circle was proportional to the number of studies involving the specific treatment. The network map for the VAS included 8 treatments, with the thickest line between Pla and Thd; the map for the SSS and FSS included 11 and 10 treatments, respectively, both with the thickest line between Pla and Prp.

Table 2 shows the results of the network meta-analysis, including all treatment strategies and their SUCRA values expressed as a percentage. Supplementary Table S3 is the rankogram that demonstrates the rank probabilities. Figure 5 presents a list of the various treatments in terms of the VAS, SSS, and FSS, in which the order is ranked by the results of SUCRA. The comparisons should be read from left to right. The estimate is located at the intersection of the column-defining treatment and the row-defining treatment.

**TABLE 2:** Summary results of all SUCRA values.

Change in the visual analog scale		Change in the symptom severity scale		Change in the functional status scale	
Therapy	SUCRA	Therapy	SUCRA	Therapy	SUCRA
Dex	79.2	Hyd	91.0	Hyd	99.5
Thd	62.7	Mld	90.1	Tld	87.4
**Prp**	60.0	Tld	74.3	**Prp**	77.1
Tld	49.2	Mhd	74.1	Soz	50.2
Bet	48.2	**Prp**	60.8	Dex	43.0
Hyd	46.2	Dsp	49.0	Dsp	41.6
Soz	24.2	Dex	36.3	Mhd	41.2
		Pir	33.8	Pir	33.9
		Thd	10.0	Mld	20.9
		Soz	2.3	Thd	6.7

#### Change in the visual analog scale

**FIGURE 4:**
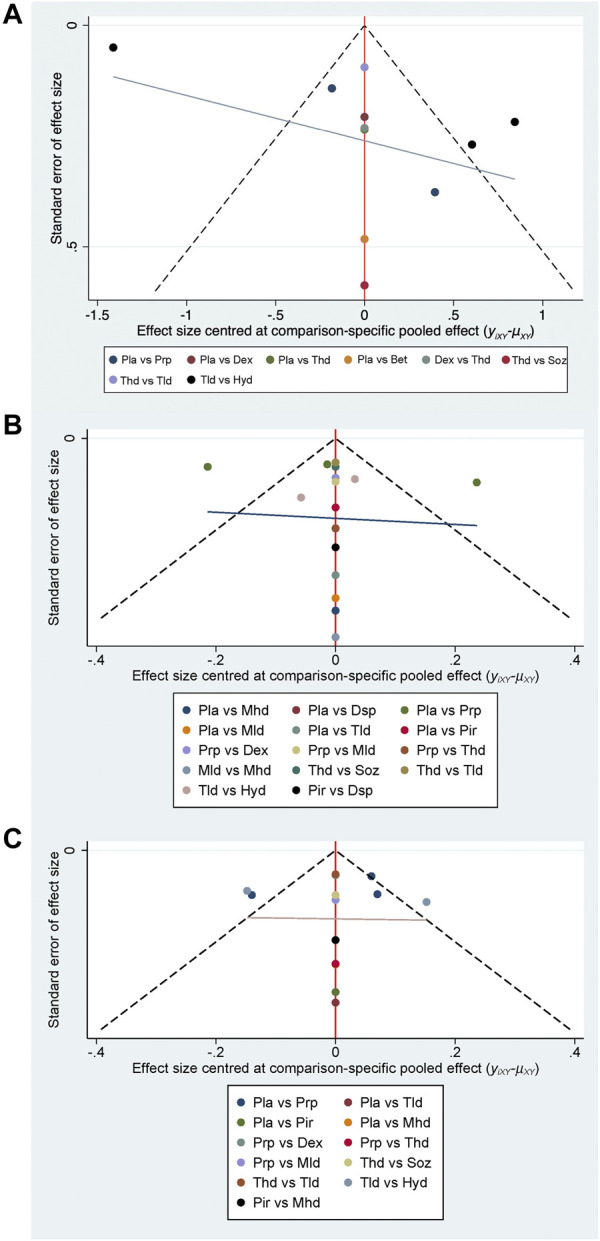
Funnel plots of the VAS **(A)**, SSS **(B),** and FSS **(C)**.

**FIGURE 5:**
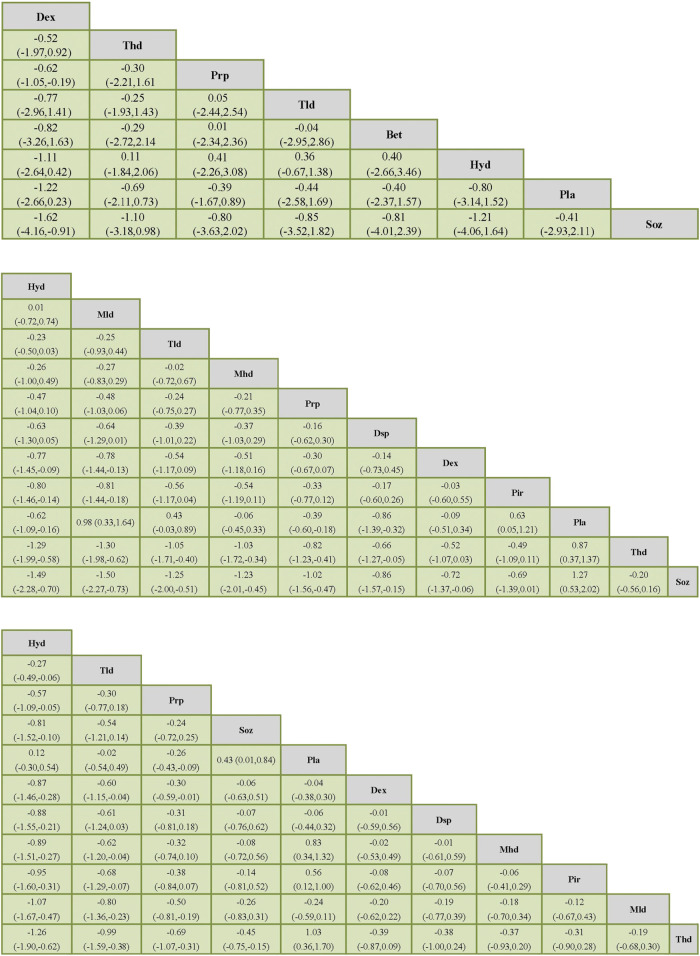
Network meta-analysis of outcomes (VAS). Network meta-analysis of outcomes (SSS). Network meta-analysis of outcomes (FSS).

Changes in the visual analog scale were available in 10 studies with 571 participants (Ginanneschi et al., 2012; Karadaş et al., 2012; Bahrami et al., 2015; Wu et al., 2017c; Dernek et al., 2017; Raeissadat et al., 2018; Wu et al., 2018; Hsu et al., 2020; Forogh et al., 2021) (Figure 5). Generally, 5% dextrose injections might be the best choice among these interventions (MD −1.22, 95% CI −2.66 to 0.23; SUCRA = 79.2%), followed by triamcinolone (high-dose) injections (MD −0.69, 95% CI −2.11 to 0.73; SUCRA = 62.7%) and PRP injections (MD −0.39, 95% CI −1.67 to 0.89; SUCRA = 60.0%). No publication bias was found in the funnel plot (See Figure 4).

#### Change in the symptom severity scale

The results of changes in the symptom severity scale were provided in 13 studies with 770 participants (Peters-Veluthamaningal et al., 2010; Atroshi et al., 2013; Bahrami et al., 2015; Uzun et al., 2017; Raeissadat et al., 2018; Güven et al., 2019; Senna et al., 2019; Shen et al., 2019; Boonhong and Thienkul, 2020b; Hsu et al., 2020; Chen et al., 2021; Forogh et al., 2021) (Figure 5). The most effective intervention was hydroxyprogesterone injections (MD −0.62, 95% CI −1.09 to −0.16; SUCRA = 91.0%), followed by methylprednisolone (low-dose) injections (MD 0.98, 95% CI 0.33 to 1.64; SUCRA = 90.1%). The SUCRA ranking of PRP was located in the fifth place (MD −0.39, 95% CI −0.60 to −0.18; SUCRA = 60.8%), and its effectiveness was second only to steroids and estrogen. No publication bias was found in the change in the symptom severity scale (See Figure 4).

#### Change in the functional status scale

Twelve studies with 659 participants provided the data on changes in the functional status scale (Peters-Veluthamaningal et al., 2010; Bahrami et al., 2015; ; Uzun et al., 2017; Raeissadat et al., 2018; Güven et al., 2019; Senna et al., 2019; Shen et al., 2019; Boonhong and Thienkul, 2020b; Hsu et al., 2020; Chen et al., 2021; Forogh et al., 2021) (Figure 5). The most effective intervention might be hydroxyprogesterone injections (MD 0.12, 95% CI −0.30 to 0.54; SUCRA = 99.5%), followed by triamcinolone (low-dose) injections (MD −0.02, 95% CI −0.54 to 0.49; SUCRA = 87.4%) and PRP injections (MD −0.26, 95% CI −0.43 to −0.09; SUCRA = 77.1%). There was no publication bias in the funnel plot (See Figure 4).

#### The effectiveness rank by SUCRA

PRP therapy was ranked third in both the VAS and FSS and fifth in the SSS by SUCRA (See Table 2). In all hormonal therapies (Hyd, Mhd, Mld, Thd, Tld, Dsp, and Bet), only Hyd and Tld were able to outperform PRP therapies both on the SSS and FSS (See Figure 5). In the SSS, PRP therapy was more effective than Dsp (MD −0.16, 95% CI −0.62 to 0.30) and Thd (MD −0.82, 95% CI −1.23 to −0.41). In the FSS, PRP was more effective than Dsp, Mhd, Mld, and Thd (MD 95% CI: −0.31 (−0.81, 0.18), −0.32 (−0.74, 0.10), −0.50 (−0.81,−0.19), and −0.69 (−1.07,−0.31)). In the VAS, PRP was more effective than Soz (MD 95% CI: −0.80 (−3.63, 2.02)) (See Supplementary Figure S4 for detail).

### Subgroup analysis and adverse events

Subgroup analysis of chronic carpal tunnel syndrome, different doses, and types of corticosteroids was not available due to the lack of data. No adverse events were reported in all 18 of the studies we included.

## Discussion

In the results section, we ranked the effectiveness of each regimen by calculating the SUCRA. Steroids and estrogen therapies were the best treatment choices (Roh et al., 2019), while PRP therapy is second only to these two. Compared with placebo, PRP therapy demonstrated better outcomes in all studies on the SSS, FSS, and VAS, suggesting the therapeutic effect of PRP for CTS. Additionally, the results of the two-by-two comparison in different indicators showed the advantage of PRP therapy compared with Thd, Mld, and Dex on the SSS and FSS (See Supplementary Figure S5). Therefore, our study indicated that PRP therapy was effective in the treatment of CTS.

Concerning efficacy, PRP therapy was second only to estrogen and steroid therapy, and it demonstrated the advantages of PRP over other therapies in the SSS and FSS. Short-term PRP treatment (within 12 weeks) also demonstrated effective outcomes. Similar conclusions had been published in other studies: Uzun et al. (2017) compared PRP with triamcinolone, and in their study, PRP provided better, but temporary, symptom relief after 3 months of treatment for CTS as no significant results were observed after 6 months; Senna et al. (2019) demonstrated that PRP injections provided significantly better results at 4 and 12 weeks for the SSS and FSS than that of methylprednisolone; and Shen et al. (2019) also supported the superiority of PRP efficacy over dextrose solution. Additionally, there was no serious complication in all the included studies of PRP treatment for CTS (Uzun et al., 2017; Raeissadat et al., 2018; Güven et al., 2019; Senna et al., 2019; Shen et al., 2019; Chen et al., 2021), whereas adverse events had been reported in steroid treatment for CTS (Wang and Carter, 2018). Moreover, Hyd therapy was optimal for both the SSS and FSS, but it could only be used for CTS in women and had several adverse effects (such as increased breast cancer risk) (Piette, 2020).

The efficacy of PRP for CTS had been reported by many authors. Malahias et al. (2018) concluded a success rate (defining a difference of more than 25% in VAS) of 76.9% for PRP treatment compared to 33.3% for placebo; Wu et al. reported that PRP treatment resulted in a considerable reduction in median nerve VAS scores, Boston Carpal Tunnel Questionnaire (BCTQ) scores, and cross-sectional area 6 months after treatment (Malahias et al., 2019); and a meta-analysis by Catapano et al. (2020) showed that PRP treatment resulted in statistically significant improvements in patients’ BCTQ. These findings were consistent with our result that PRP was a safe and effective choice to relieve pain in patients with CTS. PRP contains various growth factors from the active ingredients of degraded platelets, including the platelet-derived growth factor, transforming growth factor, epidermal growth factor, vascular endothelial growth factor, and insulin-like growth factor-1. These growth factors could improve wound healing and reduce inflammation (Sundman et al., 2011). PRP has been widely used in knee injuries, arthritis, joint pain, and inflammation (Boonhong and Thienkul, 2020a). However, the contraindications of PRP, such as pregnancy or breastfeeding, demand attention in clinical practice (Nguyen et al., 2011; Moraes et al., 2014). The most common usage was to achieve 4–7 times the concentration of platelet in clinical practice, but there was no uniform standard for its production and dosage of PRP (Dhurat and Sukesh, 2014).

Our results suggested Dex was also a promising therapeutic agent for CTS. Compared with the placebo, Dex remarkably improved patient VAS scores (95% CI: −1.22 (−2.66, 0.23)) and exerted efficacy in the patient SSS (95% CI: −0.09 (−0.51, 0.34)). Dex was recommended for patients with hypoglycemia and fluid loss, and the contraindications were glucose allergy and a history of heart disease (See Supplementary Table S5). Additionally, Soz and Pir were commonly used in CTS treatment. Soz combined with procaine injection was indicated for patients with dermatological conditions such as acne, boils, and pyoderma, while patients with acute myocardial infarction and acute alcoholic psychosis were contraindicated. Pir was recommended for patients with rheumatoid arthritis and osteoarthritis, while contraindications include bleeding disorders and stomatitis. However, Dex, Soz, and Pir had lower SUCRA ranks than PRP for the SSS and FSS in our analysis. Detailed guidelines for all the drugs are available in Supplementary Table S5.

Our study is the first network meta-analysis of PRP therapy for CTS treatment compared with other injectable drugs. We provided results not only for direct comparisons of PRP with other drug therapies but also for indirect comparisons of different therapies by network analysis. In addition, we used the SUCRA algorithm to rank the probabilities of all these treatment strategies, which could help clinicians make better choices.

There were several limitations to our study. First, there were not enough data on the VAS in the included RCTs to corroborate our conclusion. Second, no subgroup analysis was performed due to insufficient data. Additionally, the follow-up period of the included RCTs ranged from 4 weeks to 12 weeks, leading to heterogeneity in data. Moreover, no adverse event was reported in the control group of the included RCTs (see summary table for details). More RCTs are required to provide insight into the application of PRP treatment for CTS.

## Conclusion

PRP is an alternative choice for the treatment of CTS. PRP injection is second only to steroids and estrogen in the treatment of CTS, with a wide indication and safe outcome.

## Data Availability

The original contributions presented in the study are included in the article/Supplementary Material; further inquiries can be directed to the corresponding authors.

## Abbreviations

CTS, carpal tunnel syndrome; NSAID, non-steroidal anti-inflammatory drug; PRP, platelet-rich plasma; PRISMA, Preferred Reporting Items for Systematic Reviews and Meta-Analyses; RCTs, randomized controlled trials; VAS, visual analog scale; SSS, symptom severity scale; FSS, functional severity scale; CROBAT, Cochrane risk of bias assessment tool; SUCRA, surface under the cumulative ranking area; SMD, standard mean difference; MD, mean difference; Thd, triamcinolone high-dose or; Tld, triamcinolone low-dose; Prp, platelet-rich plasma; Dex, dextrose; Mhd, methylprednisolone high-dose or; Mld, methylprednisolone low-dose; Hyd, hydroxyprogesterone; Bet, betamethasone; Dsp, dexamethasone sodium phosphate; Pir, piroxicam; Soz, single ozone; BCTQ, Boston Carpal Tunnel Questionnaire.
