# Supplementary material for: Efficacy and safety of platelet-rich plasma in the treatment of carpal tunnel syndrome: A network meta-analysis of different injection treatments
Source: Front Pharmacol. 2022 Nov 10;13:906075. doi: 10.3389/fphar.2022.906075 (PMC9684083; doi:10.3389/fphar.2022.906075)
Supplement: Supplementary file 1 [file DataSheet1.doc]

**Supplemental files**

Figure S1. Heterogeneity assessment of age

Figure S2. Heterogeneity assessment of gender

Figure S3. SUCRA of VAS(a), SSS(b) and FSS(c)

Figure S4. Comparison of the efficacy of various injection therapies with PRP

Figure S5. Forest plot comparing different injection treatments

Table S1. PRISMA Checklist

Table S2. Search Strategy of Medline

Table S3. Assessment tools in Meta-analysis

Table S4. Outcome of CROBAT assessment

Table S5. Guidelines of included drugs

Figure S1. Heterogeneity assessment of age


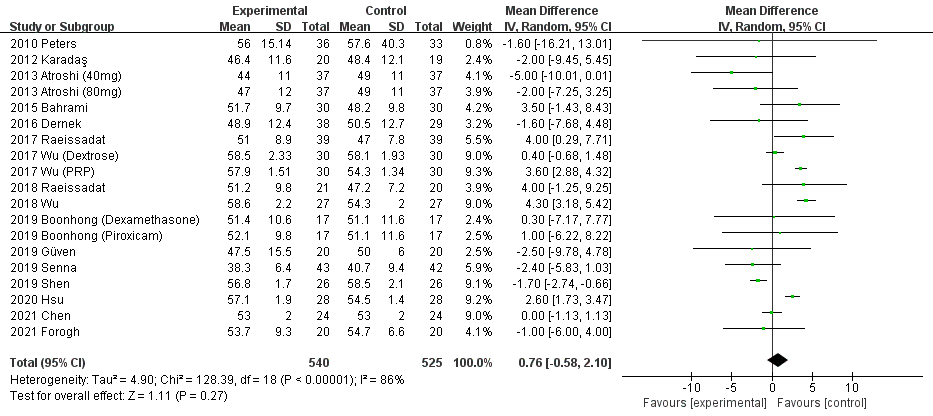


Figure S2. Heterogeneity assessment of gender


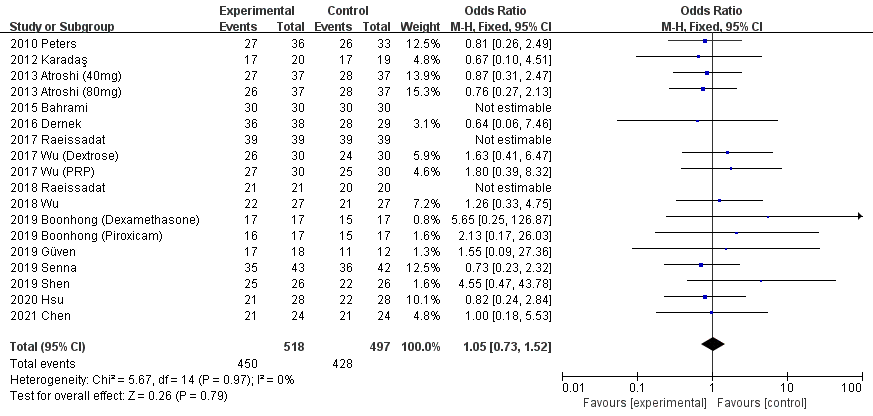


Figure S3. SUCRA of VAS(a), SSS(b) and FSS(c)

(a)


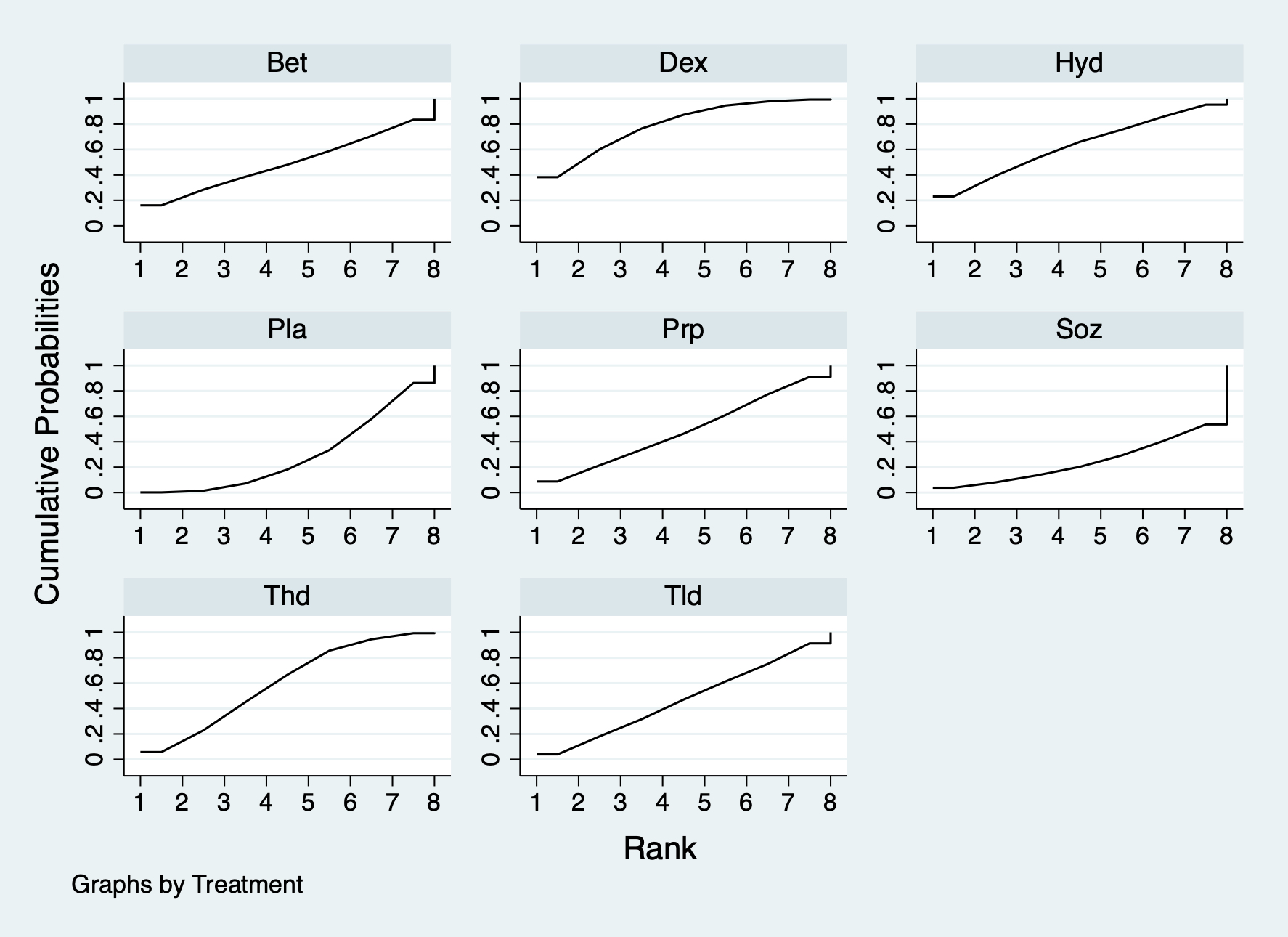


(b)


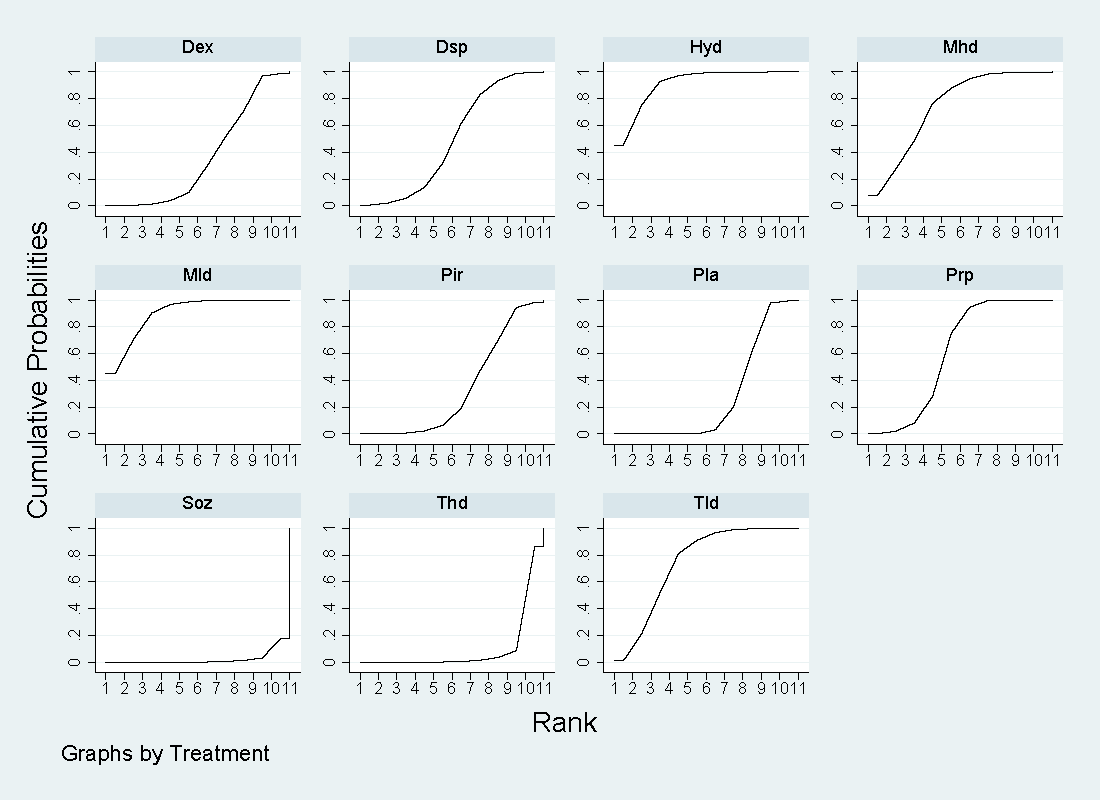


(c)


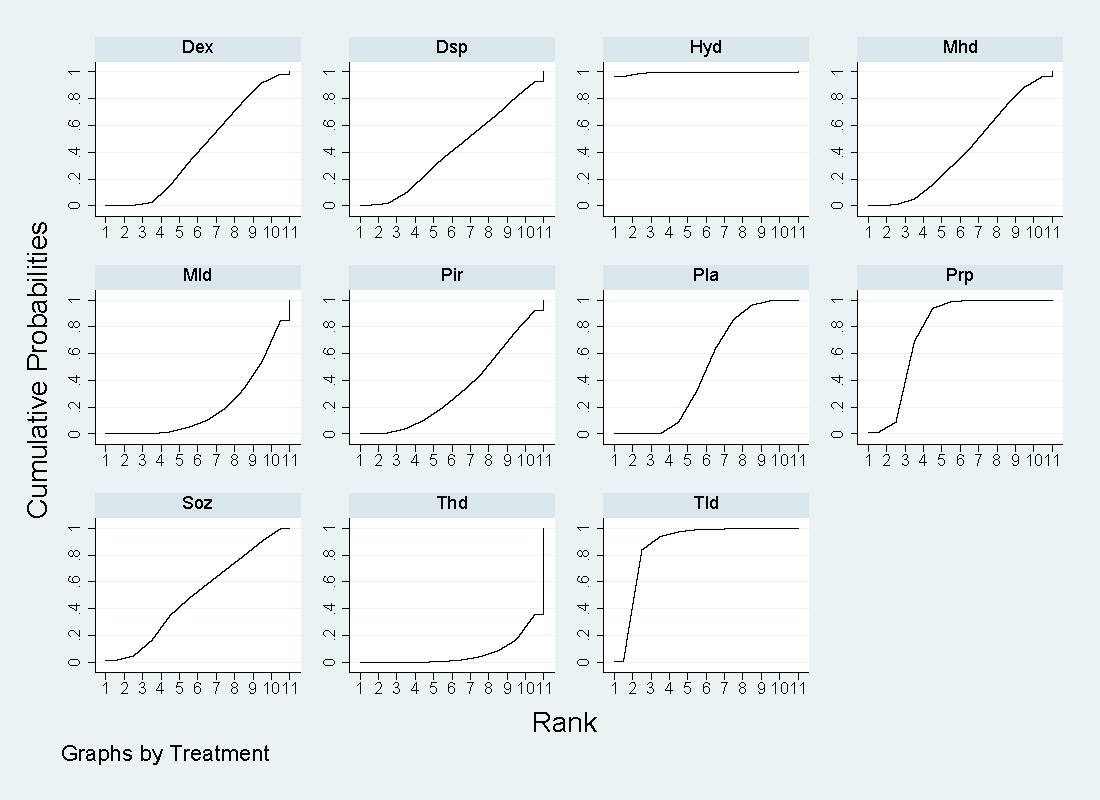


Figure S4. Comparison of the efficacy of various injection therapies with PRP

1. VAS


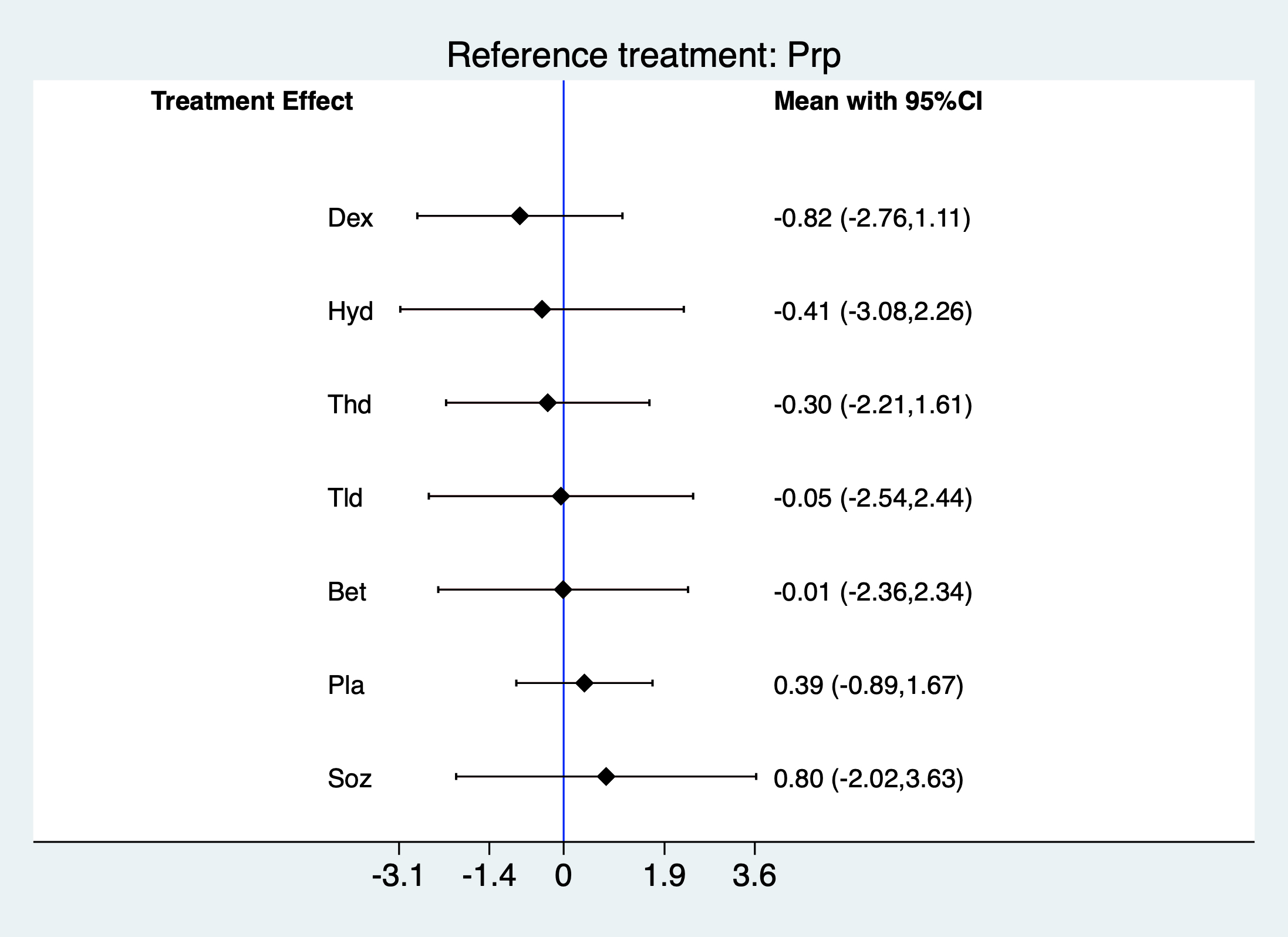


1. SSS


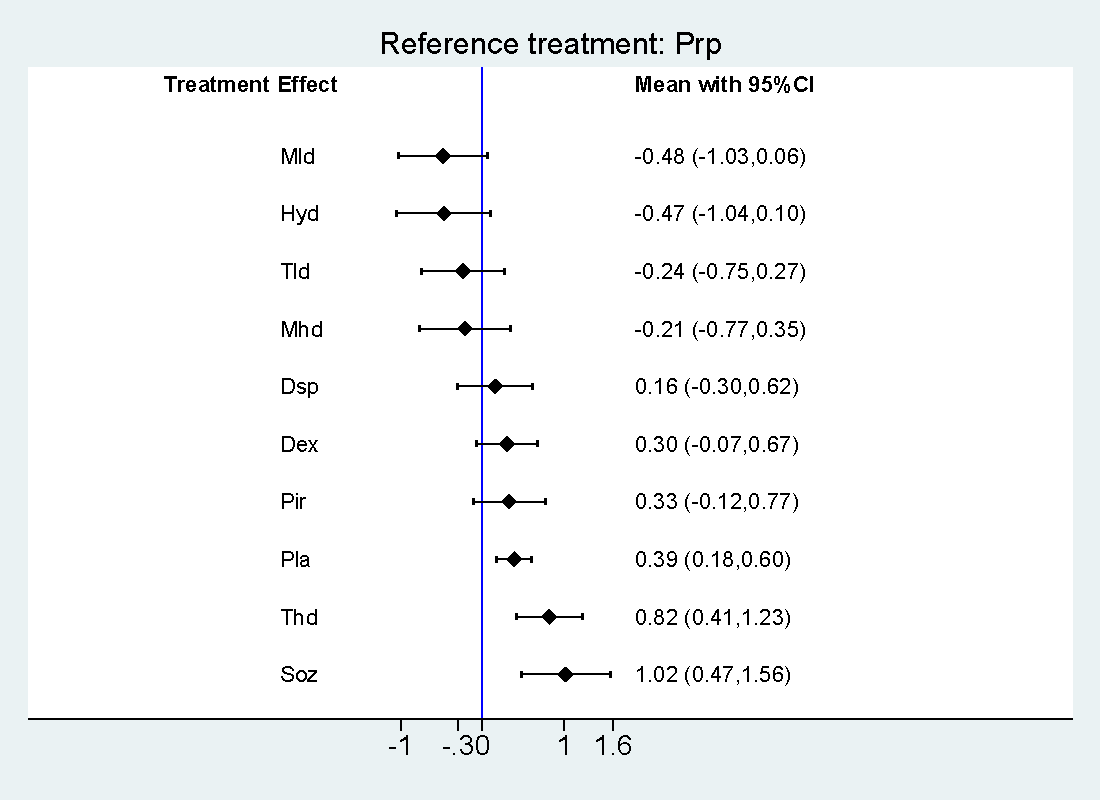


1. FSS


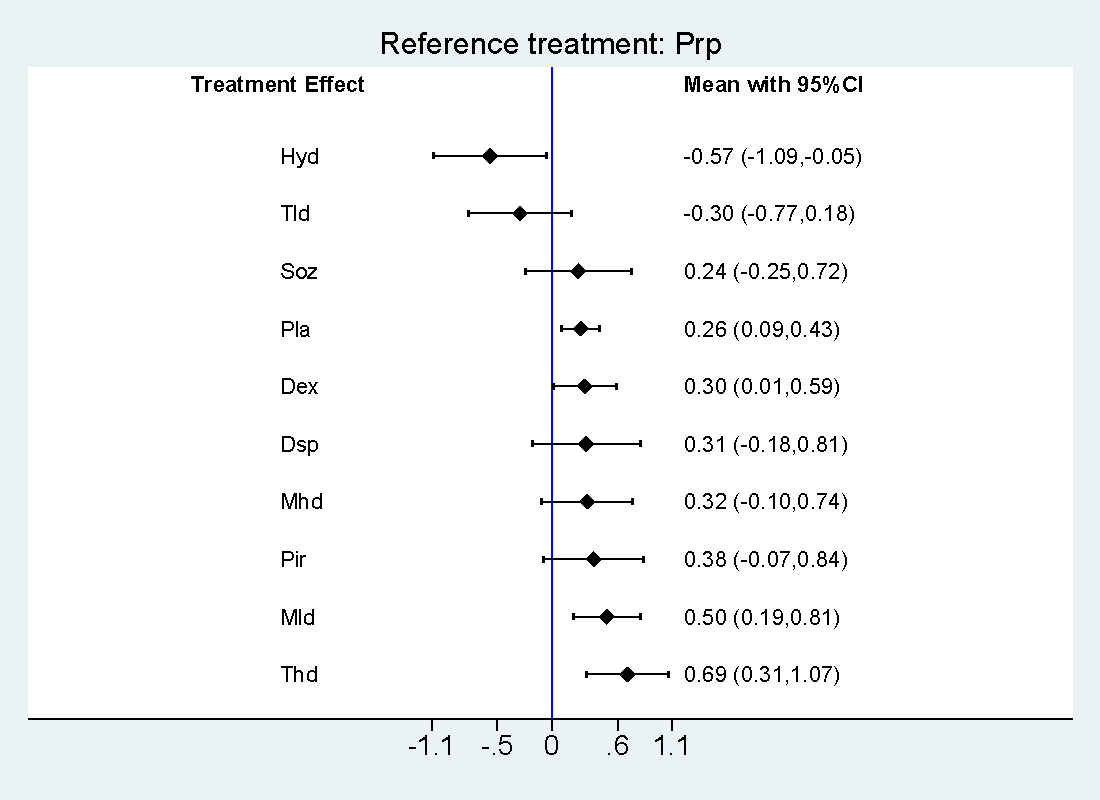


Figure S5. Forest plot comparing different injection treatments

(a) In VAS


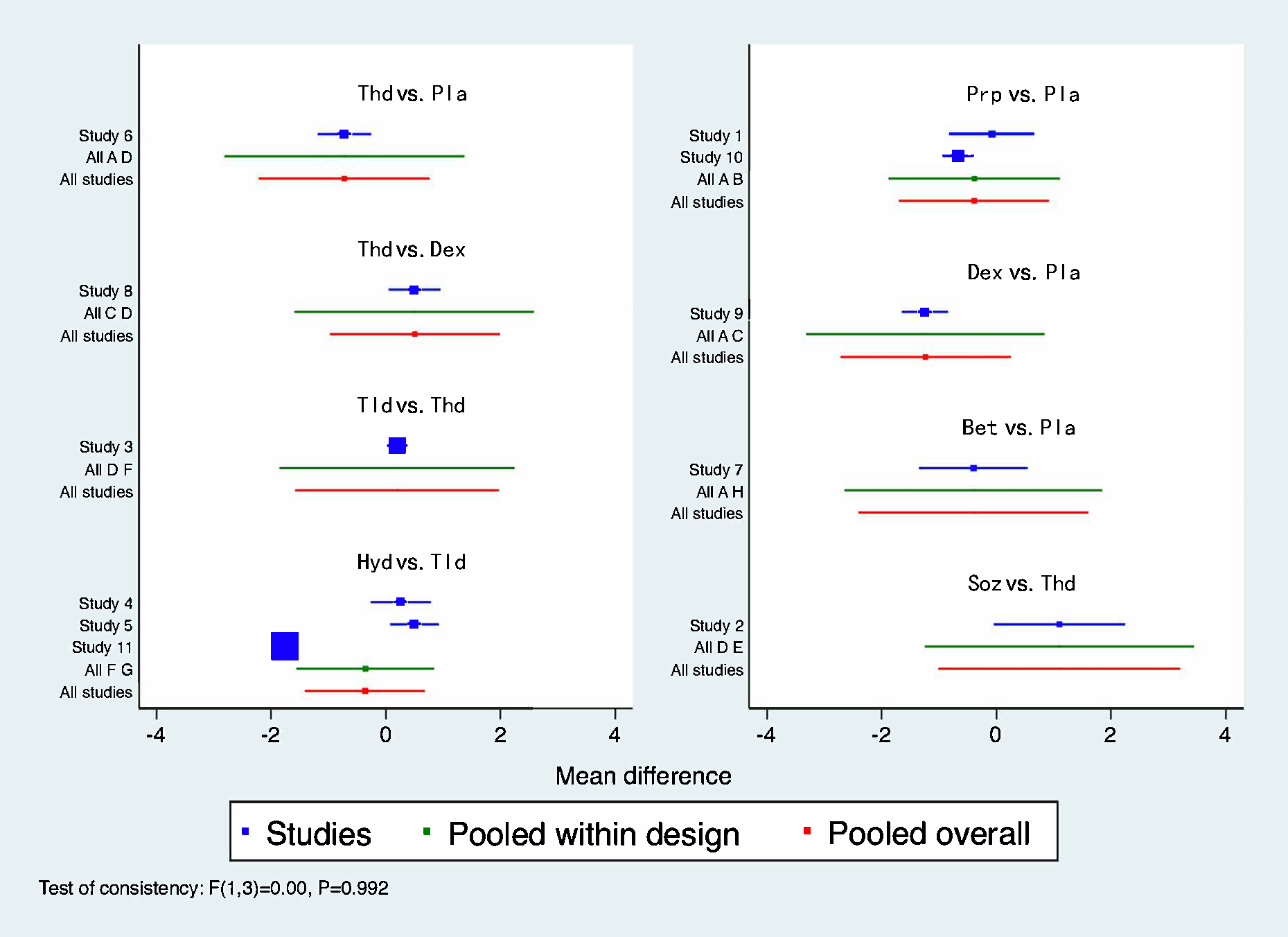


(b) In SSS


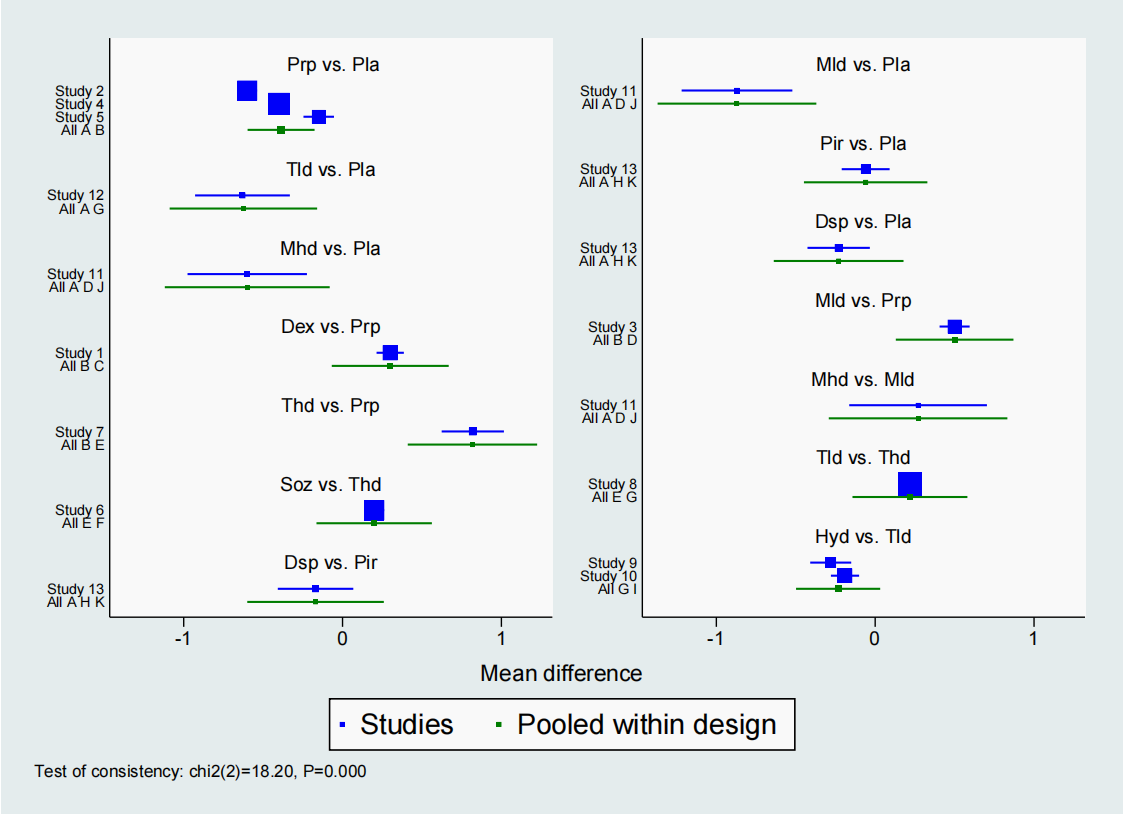


(c) In FSS


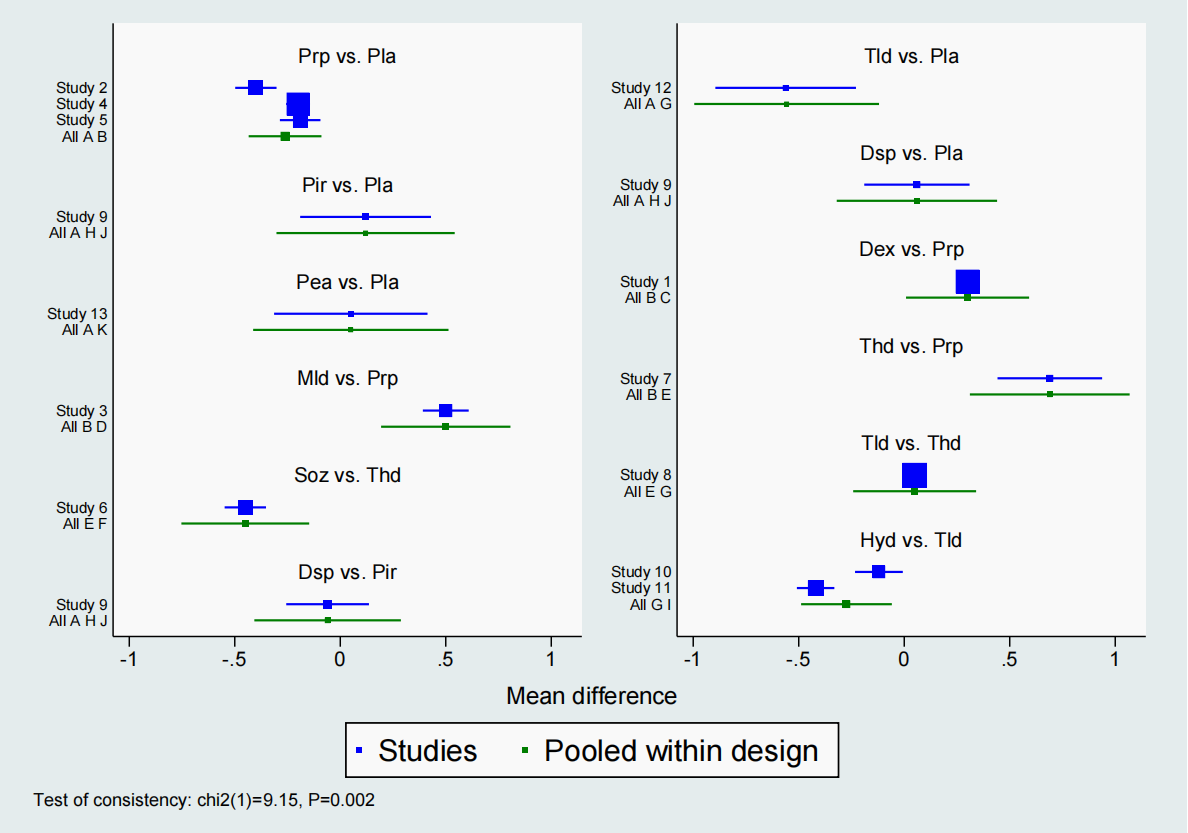


Table S1. PRISMA Checklist

| **Section/Topic** | **#** | **Checklist Item** | **Reported on Page #** |
| --- | --- | --- | --- |
| **TITLE** | | | |
| Title | 1 | Identify the report as a systematic review, meta-analysis, or both. | 1 |
| **ABSTRACT** | | | |
| Structured summary | 2 | Provide a structured summary including, as applicable: background; objectives; data sources; study eligibility criteria, participants, and interventions; study appraisal and synthesis methods; results; limitations; conclusions and implications of key findings; systematic review registration number. | 2 |
| **INTRODUCTION** | | | |
| Rationale | 3 | Describe the rationale for the review in the context of what is already known. | 5 |
| Objectives | 4 | Provide an explicit statement of questions being addressed with reference to participants, interventions, comparisons, outcomes, and study design (PICOS). | 5 |
| **METHODS** | | | |
| Protocol and registration | 5 | Indicate if a review protocol exists, if and where it can be accessed (e.g., Web address), and, if available, provide registration information including registration number. | 5 |
| Eligibility criteria | 6 | Specify study characteristics (e.g., PICOS, length of follow-up) and report characteristics (e.g., years considered, language, publication status) used as criteria for eligibility, giving rationale. | 6 |
| Information sources | 7 | Describe all information sources (e.g., databases with dates of coverage, contact with study authors to identify additional studies) in the search and date last searched. | 5 |
| Search | 8 | Present full electronic search strategy for at least one database, including any limits used, such that it could be repeated. | 5 |
| Study selection | 9 | State the process for selecting studies (i.e., screening, eligibility, included in systematic review, and, if applicable, included in the meta-analysis). | 6 |
| Data collection process | 10 | Describe method of data extraction from reports (e.g., piloted forms, independently, in duplicate) and any processes for obtaining and confirming data from investigators. | 6 |
| Data items | 11 | List and define all variables for which data were sought (e.g., PICOS, funding sources) and any assumptions and simplifications made. | 6 |
| Risk of bias in individual studies | 12 | Describe methods used for assessing risk of bias of individual studies (including specification of whether this was done at the study or outcome level), and how this information is to be used in any data synthesis. | 7 |
| **Section/Topic** | **#** | **Checklist Item** | **Reported on Page #** |
| Summary measures | 13 | State the principal summary measures (e.g., risk ratio, difference in means). | 7 |
| Synthesis of results | 14 | Describe the methods of handling data and combining results of studies, if done, including measures of consistency (e.g., I^2^) for each meta-analysis. | 7 |
| Risk of bias across studies | 15 | Specify any assessment of risk of bias that may affect the cumulative evidence (e.g., publication bias, selective reporting within studies). | 7 |
| Additional analyses | 16 | Describe methods of additional analyses (e.g., sensitivity or subgroup analyses, meta-regression), if done, indicating which were pre-specified. | 7 |
| **RESULTS** | | | |
| Study selection | 17 | Give numbers of studies screened, assessed for eligibility, and included in the review, with reasons for exclusions at each stage, ideally with a flow diagram. | 8 |
| Study characteristics | 18 | For each study, present characteristics for which data were extracted (e.g., study size, PICOS, follow-up period) and provide the citations. | 8 |
| Risk of bias within studies | 19 | Present data on risk of bias of each study and, if available, any outcome level assessment (see item 12). | 10 |
| Results of individual studies | 20 | For all outcomes considered (benefits or harms), present, for each study: (a) simple summary data for each intervention group (b) effect estimates and confidence intervals, ideally with a forest plot. | 10 |
| Synthesis of results | 21 | Present the main results of the review. If meta-analyses done, include for each, confidence intervals and measures of consistency. | 10 |
| Risk of bias across studies | 22 | Present results of any assessment of risk of bias across studies (see Item 15). | 10 |
| Additional analysis | 23 | Give results of additional analyses, if done (e.g., sensitivity or subgroup analyses, meta-regression [see Item 16]). | 18 |
| **DISCUSSION** | | | |
| Summary of evidence | 24 | Summarize the main findings including the strength of evidence for each main outcome; consider their relevance to key groups (e.g., healthcare providers, users, and policy makers). | 18 |
| Limitations | 25 | Discuss limitations at study and outcome level (e.g., risk of bias), and at review-level (e.g., incomplete retrieval of identified research, reporting bias). | 23 |
| Conclusions | 26 | Provide a general interpretation of the results in the context of other evidence, and implications for future research. | 23 |
| **FUNDING** | | | |
| Funding | 27 | Describe sources of funding for the systematic review and other support (e.g., supply of data); role of funders for the systematic review. | 24 |

*From:* Moher D, Liberati A, Tetzlaff J, Altman DG, The PRISMA Group (2009). Preferred Reporting Items for Systematic Reviews and Meta-Analyses: The PRISMA Statement. PLoS Med 6(6): e1000097. doi:10.1371/journal.pmed1000097

For more information, visit: **www.prisma-statement.org**.

Table S2. Search Strategy of Medline

| Procedure | Strategy | Result^a^ |
| --- | --- | --- |
| 1 | exp carpal tunnel syndrome/ or carpal tunnel stenosis/ | 9222 |
| 2 | (carpal tunnel syndrome* or carpal tunnel stenosis*).ti.ab | 9345 |
| 3 | 1 or 2 | 11787 |
| 4 | (clinical trial or randomized controlled trial).pt. | 932733 |
| 5 | 3 and 4 | 797 |

^a^Search date: Oct 17^th^, 2022

(carpal tunnel syndrome[MeSH Terms] OR carpal tunnel stenosis[MeSH Terms] OR carpal tunnel syndrome[Title/Abstract] OR carpal tunnel stenosis[Title/Abstract]) AND (clinical trial[Publication Type] OR randomized controlled trial[Publication Type])

Table S3. Assessment tools in Meta-analysis

1. **Cochrane Risk Of Bias Assessment Tool (CROBAT)**

| Random sequence generation | Allocation concealment | Blinding of participants and personnel | Blinding of outcome assessment | Incomplete outcome data | Selective reporting | Other bias |
| --- | --- | --- | --- | --- | --- | --- |
|  |  |  |  |  |  |  |
|  |  |  |  |  |  |  |

Each question had 3 answers: “Low risk”, “Moderate” and “High risk”.

“Low risk” when detailed methods were founded in manuscript.

“Moderate” when declared blinded without detailed method.

“High risk” when found no relevant declaration.

1. **Grading of Recommendations, Assessment, Development and Evaluation (GRADE)**

| Risk of  Bias | Inconsistency | Indirectness | Imprecision | Publication bias | Plausible Confounding | Magnitude of effect | Dose-response gradient |
| --- | --- | --- | --- | --- | --- | --- | --- |
|  |  |  |  |  |  |  |  |
|  |  |  |  |  |  |  |  |

Each following question had 3 answers: “No serious risk”, “Serious risk” and “Very serious risk”.

Risk of Bias: “Serious risk” when sensitive analysis resulted in significant difference.

Inconsistency: “No serious risk” when I^2^≤50%; “Serious risk”when 50<I^2^≤75%; “Very serious risk” when I^2^>75%

Indirectness: Comprehensive consideration in combination with information.

Imprecision: “Serious risk” when P>0.05

Publication bias: “No serious risk” when Egger’s test P>0.05; “Serious risk”when 0.01<P≤0.05; “Very serious risk” when P≤0.01.

Plausible Confounding had 2 answers: “No” and “Yes”. It would be assessed by comprehensive consideration in combination with information.

Magnitude of effect had 3 answers: “No”, “Yes” and “Extremely” related to odds ratio (OR). “No” when 0.5<OR<2; “Yes” when 0.2<OR≤0.5 or 2≤OR<5; “Extremely” when OR≤0.2 or OR≥5.

Dose-response gradient had 2 answers: “No” and “Yes”. “Yes” when P value of dose-response related outcome ≤0.05.

There were 4 levels of quality: “High”, “Moderate”, “Low” and “Very low”. Evidence of RCTs were initially assessed as “High”. “Serious risk” would reduce 1 level of quality and “Very serious risk” would reduce 2 levels of quality. While “Yes” could promote 1 level of quality and “Extremely” could promote 2 level of quality.

Table S4. Outcome of CROBAT assessment

| Author | Trail Name | Random sequence generation | Allocation concealment | Blinding of participant and personnel | Blinding of outcome assessment | Incomplete outcome data | Selective reporting | Other bias |
| --- | --- | --- | --- | --- | --- | --- | --- | --- |
| Chen 2021 | NCT03184688 | Randomly assigned on a 1:1 basis | Unclear | Yes | Yes | No | Unclear | No |
| Forogh 2021 | IRCT20151017024572N5 | Random assignment but it is not clear exactly how it is assigned | Random drug dispensing using sealed envelopes | Yes | Yes | No | No | No |
| Hsu 2020 | NCT03072290 | Randomly assigned by a random number generator | Random drug dispensing using sealed envelopes | Yes | Yes | No | No | No |
| Boonhong 2019 | RA 57/114 | Randomly assigned by a random number generator | Random drug dispensing using sealed envelopes | Yes | Yes | No | Unclear | No |
| Güven 2019 | E‐14‐267 | The study is lack of randomization | The study is lack of randomization | Yes | Yes | No | Unclear | No |
| Senna 2019 | NCT03863873 | Randomly allocated by means of block randomization | Random drug dispensing using sealed envelopes | Yes | Yes | No | Unclear | No |
| Shen 2019 | NCT02696161 | Randomly assigned on a 1:1 basis through computer randomization | Random assignment but it is not clear exactly how it is assigned | Yes | Yes | No | Unclear | No |
| Raeissadat 2018 | IRCT2017041513442N13 | Randomly allocated through online randomization website (https://www.randomizer.org) | Random drug dispensing using sealed envelopes | Yes | Yes | No | No | No |
| Wu 2018 | NCT02990962 | Randomly assigned on a 1:1 basis | Random drug dispensing using sealed envelopes | Yes | Yes | No | No | No |
| Raeissadat 2017 | N/A^*^ | Randomly assigned by a random number generator | Random drug dispensing using sealed envelopes | Yes | Yes | No | No | No |
| Uzun 2017 | N/A | The study is lack of randomization | The study is lack of randomization | No | Yes | No | No | No |
| Wu 2017 | NCT02539186 | Randomly assigned on a 1:1 basis | Random drug dispensing using sealed envelopes | Yes | Yes | No | No | No |
| Wu 2017 | NCT02809261 | Randomly assigned on a 1:1 basis | Random drug dispensing using sealed envelopes | Yes | Yes | No | No | No |
| Dernek 2016 | N/A | The study is lack of randomization | The study is lack of randomization | Yes | Yes | No | Unclear | No |
| Bahrami 2015 | IRCT2013101313442N4 | Randomly assigned by simple random sampling | Random drug dispensing using sealed envelopes | Yes | Yes | No | No | No |
| Atroshi 2013 | NCT00806871 | Randomly assigned on a 1:1:1 basis | Randomly assigned on a 1:1:1 basis | Yes | Yes | No | Unclear | No |
| Karadaş 2012 | N/A | Random assignment but it is not clear exactly how it is assigned | Random assignment but it is not clear exactly how it is assigned | Yes | Yes | No | Unclear | No |
| Ginanneschi 2012 | N/A | Random assignment but it is not clear exactly how it is assigned | Unclear | Yes | Yes | No | Unclear | No |
| Peters-Veluthamaningal 2010 | N/A | Randomly allocated through online randomization website (https://www.randomizer.org) | Random drug dispensing using sealed envelopes | Yes | Yes | No | No | No |

*N/A = not applicable

Table S5. Guidelines of included drugs

| Drugs | Recommended dose | Injection method | Indications | Contraindications |
| --- | --- | --- | --- | --- |
| Platelet-rich plasma[1] | The dose is flexible, some articles report that PRP can be made with less than 10 ml of whole blood, even 3.5 ml; on the contrary, it can also be made with a large amount of whole blood; the effect is 4-7 times the original platelet concentration | Ultrasound-guided injection | PRP therapy can be used to treat many conditions including knee injuries, arthritis, joint pain and inflammation, back injuries, and bodily pain. | History of metastatic conditions like cancer or disease; Possibility of infections in the areas targeted for PRP therapy; Pregnancy or breastfeeding; History of metastatic conditions like cancer or disease; corticosteroid injections and NSAIDS; History of severe liver disease |
| Dextrose(5%)[2,3] | 5 cc of 5% dextrose | Perineural injection | Dextrose can be used to treat low blood sugar; add fluid to the body after fluid loss, to mix with certain drugs that are given as an injection, or to add calories to a total parenteral nutrition | Have an allergic reaction to dextrose; diabetes (unless you are using dextrose to treat insulin-induced hypoglycemia); History of heart disease, coronary artery disease, or a stroke; asthma; kidney disease; a possible head injury; alcoholism |
| Methylprednisolone[4,5] | The adult dosage is mainly 40mg each time. The specific dose selection depends on the situation in clinical trials | Ultrasound-guided injection | Methylprednisolone can be used to treat many different inflammatory conditions such as arthritis, lupus, psoriasis, ulcerative colitis, allergic disorders, gland (endocrine) disorders, and conditions that affect the skin, eyes, lungs, stomach, nervous system, or blood cells; Methylprednisolone may also be used for purposes not listed in this guide. | Have a fungal infection anywhere in body; a thyroid disorder; History of herpes infection of the eyes;  stomach ulcers, ulcerative colitis, or diverticulitis; depression, mental illness, or psychosis; liver disease (especially cirrhosis); high blood pressure; osteoporosis; a muscle disorder such as myasthenia gravis; or multiple sclerosis. |
| Triamcinolone[6,7] | 40mg each time | Ultrasound-guided injection | Triamcinolone oral (taken by mouth) is used to treat many different conditions such as allergic disorders, skin conditions, ulcerative colitis, arthritis, lupus, psoriasis, or breathing disorders; Triamcinolone may also be used for purposes not listed in this guide. | Have a fungal infection anywhere in body or have a allergy to triamcinolone; liver disease (such as cirrhosis); History of kidney disease; a thyroid disorder; diabetes; a history of malaria; tuberculosis; osteoporosis; a muscle disorder such as myasthenia gravis; glaucoma or cataracts; herpes infection of the eyes; stomach ulcers, ulcerative colitis, or diverticulitis; depression or mental illness; congestive heart failure; or high blood pressure |
| Single ozone (O2-O3)[8] | 30 μg | Mixed with lidocaine for injection | Ozone therapy can be used to treat skin diseases, such as acne, boils, pyoderma, herpes; it can also be used for cosmetic treatments, such as telangiectasia, scarring. | Ozone therapy is contraindicated in persons suffering from the following diseases: acute phase of myocardial infarction, acute alcoholic psychosis (alcohol intoxication), convulsive syndrome, acute pancreatitis, hyperthyroidism, arterial hypotension, hypoglycemia, hypocalcemia, thrombocytopenia, internal bleeding. |
| Piroxicam[9] | 10 mg or 20 mg; no more than 30-40 mg/d | Injection | Rheumatoid arthritis, osteoarthritis | Bleeding disorders, duodenal/gastric/peptic ulcer, stomatitis, SLE, ulcerative colitis, upper GI disease, late pregnancy. |
| Hydroxy progesterone[10] | 500 mg once | Local injection | Hydroxyprogesterone is used for contraception, functional uterine bleeding, dysmenorrhea, premenstrual tension, endometriosis, habitual abortion, evaluation of ovarian function and diagnosis of pregnancy, suppression of postpartum lactation, endometrial cancer; can be used as a chemotherapy drug for tumor diseases. | Hydroxyprogesterone caproate is contraindicated in patients with known or suspected carcinoma of the breast, other hormone-sensitive cancer, or history of these conditions; undiagnosed abnormal vaginal bleeding; liver dysfunction or disease; missed abortion, and in those with a history of hypersensitivity to the drug. |
| Dexamethasone sodium phosphate[11] | 60 mg | Ultrasound-guided injection | Dexamethasone is used for many health problems like allergy signs, asthma, adrenal gland problems, blood problems, skin rashes, or swelling problems. | Contraindications of dexamethasone include, but are not limited to: uncontrolled infections; known hypersensitivity to dexamethasone; cerebral malaria; systemic fungal infection; concurrent treatment with live virus vaccines (including smallpox vaccine) |
| Betamethasone[12] | The usual betamethasone dose ranges from 0.25 to 9 mg | Injection | Betamethasone is used to treat many different inflammatory conditions such as allergic reactions, ulcerative colitis, arthritis, lupus, multiple sclerosis, inflammation of joints or tendons, and problems caused by low adrenal hormone levels | Idiopathic thrombocytopenic purpura (ITP); History of heart disease, hypertension; thyroid disorders; parasitic infections that cause diarrhea (e.g., nematodes)); herpes infection of the eye; muscle diseases, such as myasthenia gravis; kidney disease ; cirrhosis of the liver or other liver diseases; mental illness or psychosis; gastric ulcer, diverticulitis, colostomy or ileostomy; perforation (hole or tear) in the stomach or intestines; malaria; or osteoporosis or low bone mineral density |

**Reference**

1. Dhurat R, Sukesh M. Principles and Methods of Preparation of Platelet-Rich Plasma: A Review and Author's Perspective. J Cutan Aesthet Surg. 2014 Oct-Dec;7(4):189-97. doi: 10.4103/0974-2077.150734. PMID: 25722595; PMCID: PMC4338460.
2. Lin CP, Chang KV, Huang YK, Wu WT, Özçakar L. Regenerative Injections Including 5% Dextrose and Platelet-Rich Plasma for the Treatment of Carpal Tunnel Syndrome: A Systematic Review and Network Meta-Analysis. Pharmaceuticals (Basel). 2020 Mar 18;13(3):49.
3. Wu YT, Ho TY, Chou YC, Ke MJ, Li TY, Tsai CK, Chen LC. Six-month Efficacy of Perineural Dextrose for Carpal Tunnel Syndrome: A Prospective, Randomized, Double-Blind, Controlled Trial. Mayo Clin Proc. 2017 Aug;92(8):1179-1189.
4. Lyon C, Syfert J, Nashelsky J. Clinical Inquiry: Do corticosteroid injections improve carpal tunnel syndrome symptoms? J Fam Pract. 2016 Feb;65(2):125-8.
5. Nair PP, Wadwekar V, Chakkalakkoombil SV, Narayan SK, Marusani R, Murgai A, Thirunavukkarasu S, Krishnamoorthy A, Thazhath HK. Comparison of proximal and distal corticosteroid injections for carpal tunnel syndrome. Muscle Nerve. 2020 Jul;62(1):89-94.
6. Hsu PC, Liao KK, Lin KP, Chiu JW, Wu PY, Chou CL, Wang NY, Wang JC. Comparison of Corticosteroid Injection Dosages in Mild to Moderate Idiopathic Carpal Tunnel Syndrome: A Randomized Controlled Trial. Arch Phys Med Rehabil. 2020 Nov;101(11):1857-1864.
7. Wipperman J, Goerl K. Carpal Tunnel Syndrome: Diagnosis and Management. Am Fam Physician. 2016 Dec 15;94(12):993-999.
8. Forogh B, Mohamadi H, Fadavi HR, Madani SP, Aflakian N, Ghazaie F, Babaei-Ghazani A. Comparison of Ultrasound-Guided Local Ozone (O2-O3) Injection Versus Corticosteroid Injection in Patients With Mild to Moderate Carpal Tunnel Syndrome. Am J Phys Med Rehabil. 2021 Feb 1;100(2):168-172.
9. Boonhong J, Thienkul W. Effectiveness of Phonophoresis Treatment in Carpal Tunnel Syndrome: A Randomized Double-blind, Controlled Trial. PM R. 2020 Jan;12(1):8-15.
10. Bahrami MH, Shahraeeni S, Raeissadat SA. Comparison between the effects of progesterone versus corticosteroid local injections in mild and moderate carpal tunnel syndrome: a randomized clinical trial. BMC Musculoskelet Disord. 2015 Oct 26;16:322.
11. Boonhong J, Thienkul W. Effectiveness of Phonophoresis Treatment in Carpal Tunnel Syndrome: A Randomized Double-blind, Controlled Trial. PM R. 2020 Jan;12(1):8-15.
12. Dernek B, Aydin T, Koseoglu PK, Kesiktas FN, Yesilyurt T, Diracoglu D, Aksoy C. Comparison of the efficacy of lidocaine and betamethasone dipropionate in carpal tunnel syndrome injection. J Back Musculoskelet Rehabil. 2017;30(3):435-440.
